# Supplementary material for: ENO2 as a Biomarker Regulating Energy Metabolism to Promote Tumor Progression in Clear Cell Renal Cell Carcinoma
Source: Biomedicines. 2023 Sep 9;11(9):2499. doi: 10.3390/biomedicines11092499 (PMC10525605; doi:10.3390/biomedicines11092499)
Supplement: Supplementary file 1 [file biomedicines-11-02499-s001.zip › biomedicines-2533838-supplementary.pdf]

Supplementary table

Supplementary Table S1. Univariate and multivariate analyses of ENO2 mRNA level and patient disease–free survival (DFS).

	Univariate analysis			Multivariate analysis[c]		
Variable	HR[a]	95%CI[b]	P	HR	95% CI	P
Disease–free survival						
Age (years)						
≤60 (n = 238)	1.468	0.930-2.318	0.099			
>60 (n = 198)						
Gender						
Female (n = 149)	1.144	0.699-1.873	0.591			
Male (n = 287)						
T stage						
T1 or T2 (n = 292)	6.556	3.923-10.956	0.000*			
T3 or T4 (n = 144)						
N stage						
N0 (n = 192)	7.098	3.212-15.687	0.000*	3.319	1.490-7.392	0.003*
N1 (n = 13)						
M stage						
M0 (n = 354)	11.937	7.501-18.996	0.000*	3.457	1.797-6.650	0.000*
M1 (n = 53)						
G grade						
G1 or G2 (n = 210)	5.899	3.109-11.195	0.000*	2.133	1.000-4.549	0.050*
G3 or G4 (n = 219)						
TNM stage						
I+II (n = 279)	11.458	6.173-21.270	0.000*	4.501	1.743-11.362	0.002*
III+IV (n = 115)						
ENO2						
Low(n = 225)	2.175	1.348-3.510	0.001*			
High (n = 211)						

a Hazard ratio, estimated from Cox proportional hazard regression model.

b Confidence interval of the estimated HR.

c Multivariate models were adjusted for N, M classification, G stage and TNM stage.

Supplementary Table S2. Correlation between ENO2 mRNA expression and clinicopathological parameters of ccRCC patients.

Parameter		Number	ENO2 mRNA expression		P value
			Low	High	
Age(years)	<=60	267	138	129	
	>60	270	129	141	0.365
gender	female	191	112	79	
	male	346	155	191	0.002*
T stage	T1+T2	344	187	157	
	T3+T4	193	80	113	0.004*
N stage	N0	239	125	114	
	N1	17	5	12	0.068
M stage	M0	425	219	206	
	M1	79	34	45	0.166
TNM stage	I+II	326	181	145	
	III+IV	208	85	123	0.001*
Disease	DiseaseFree	310	173	137	
Free Status	Recurred/Progressed	128	53	75	0.006*

Supplementary figure

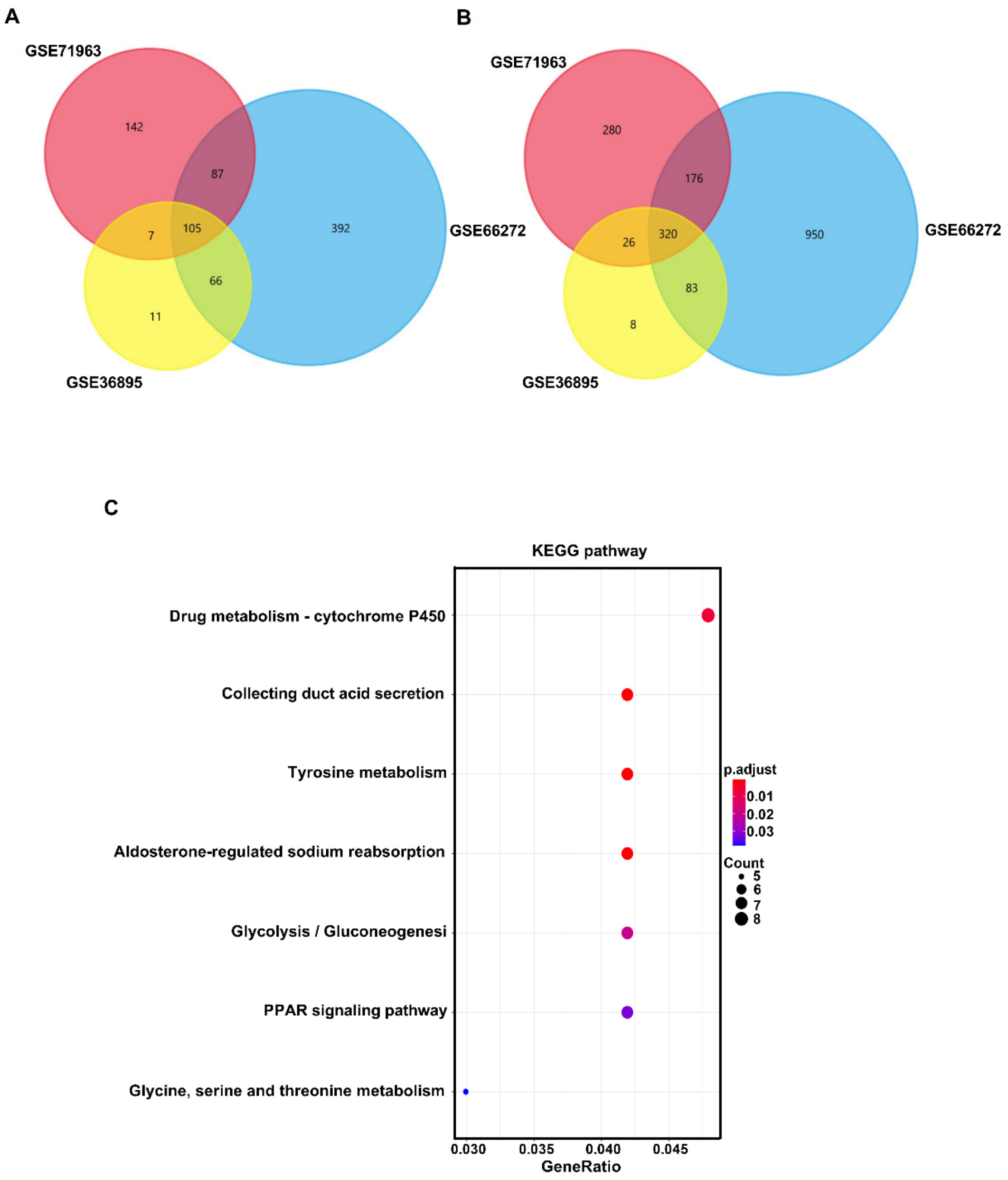

Supplemental Figure S1. Identifying differentially expressed genes

Identify the DEGs of three ccRCC gene expression profile data GSE36895, GSE66272, GSE71963, $p < 0.05$ and $|FC| > 2$ are the screening criteria used .(A) 105 up-regulated DEGs in ccRCC. (B) 420 down-regulated DEGs in ccRCC. (C) Analysis of the down-regulated KEGG pathway, p value<0.05 is considered a significant difference.

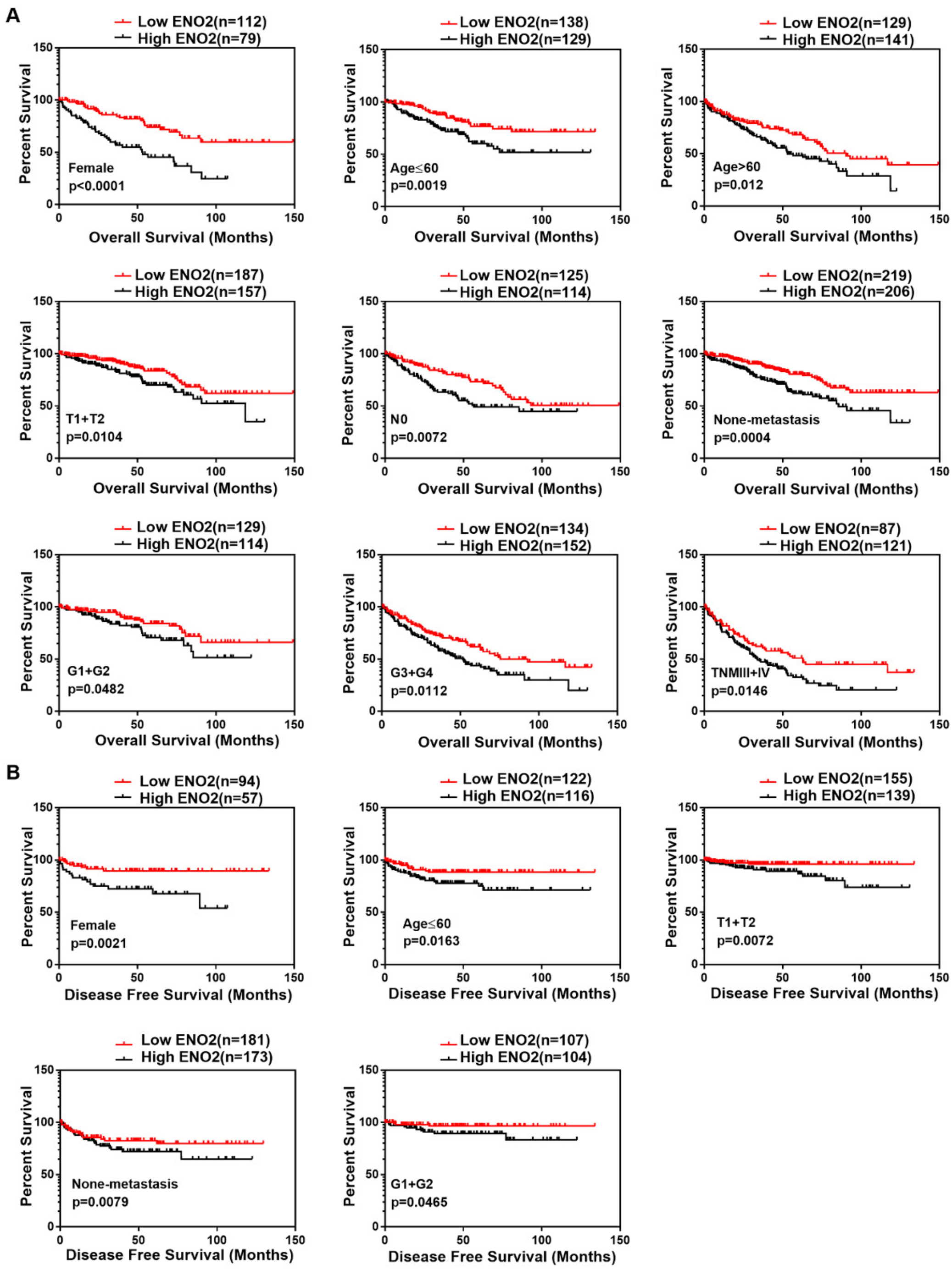

Supplemental Figure S2. ENO2 is overexpressed and indicates poor survival in clear cell renal cell carcinoma

(A) An analysis of Kaplan-Meier curves stratified by subgroups to determine overall survival. The p value is obtained through Log-rank (Mantel-Cox) test. (B) An analysis of Kaplan-Meier curves stratified by subgroups to determine disease-free survival. The p value is obtained through Log-rank (Mantel-Cox) test.

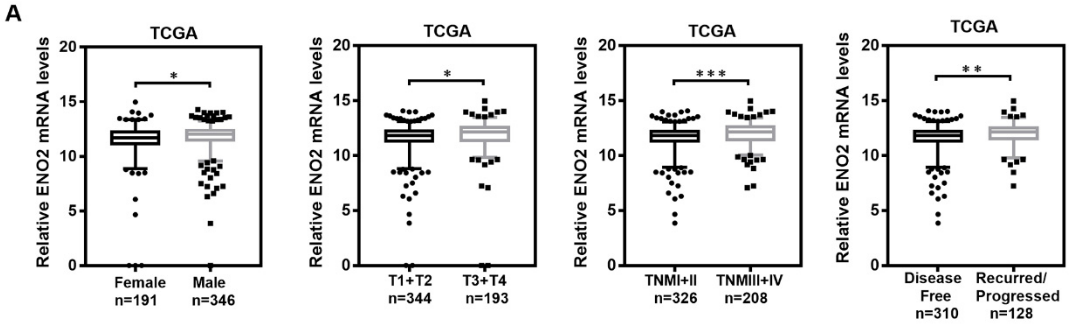

Supplemental Figure S3. ENO2 expression correlates with clinicopathological parameters of clear cell renal cell carcinoma

(A) The correlations between ENO2 expression and different clinicopathological parameters based on the data from the TCGA-KIRC database; t-test, **** P <0.0001, *** P <0.001, ** P <0.01, and * P <0.05 (Independent-Samples t-test for statistics).

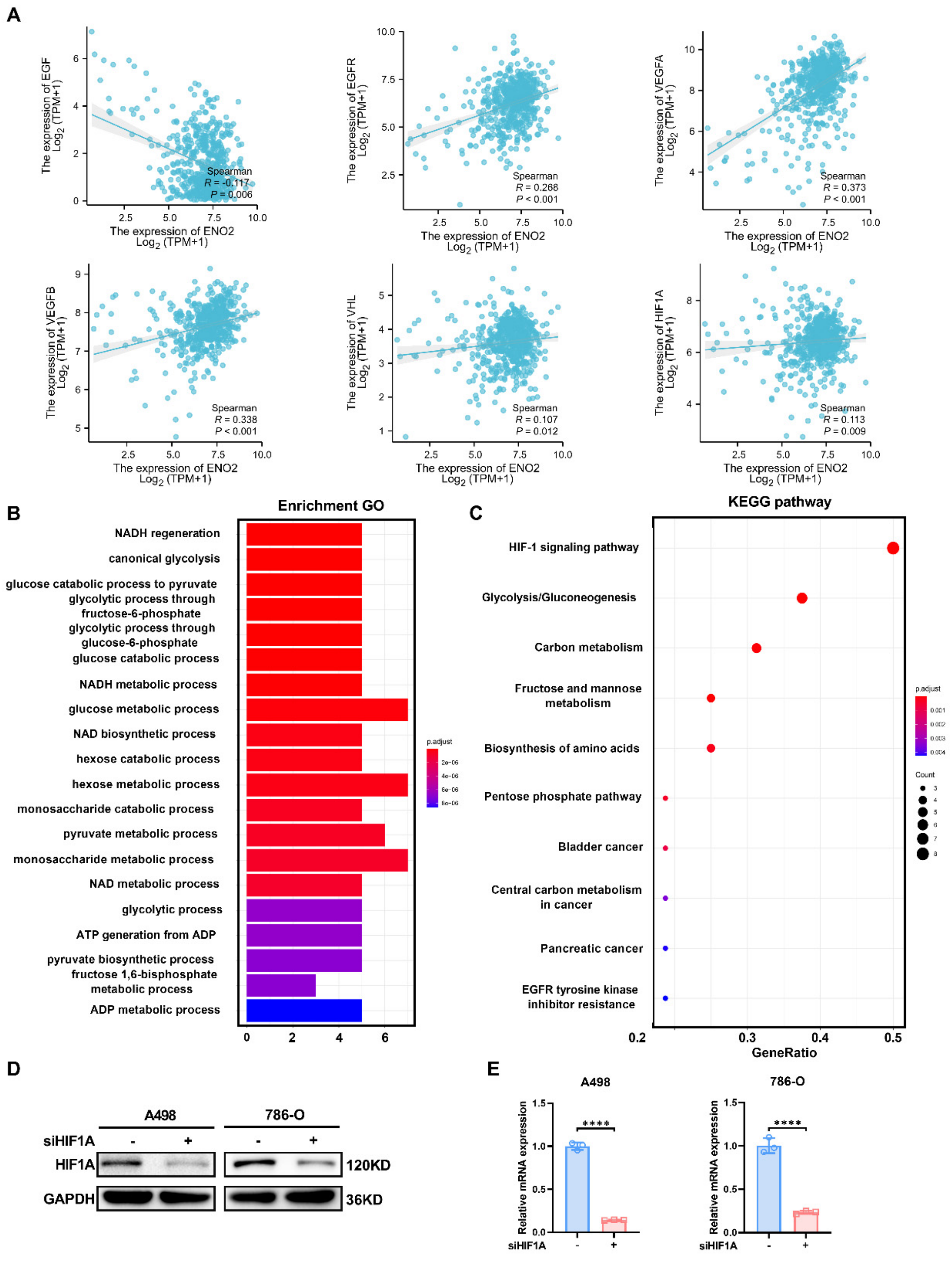

Supplemental Figure S4. ENO2 is significantly related to important genes in ccRCC (A) The correlation graph between ENO2 and EGF, EGFR, VEGFA, VEGFB, VHL, HIF1A expression based on the data from the TCGA-KIRC database. (B) GO annotations for PPI network. (C) KEGG pathway analysis for PPI network. (D) Western blot analysis of HIF1A knockdown. (E) qRT-PCR results of HIF1A knockdown.

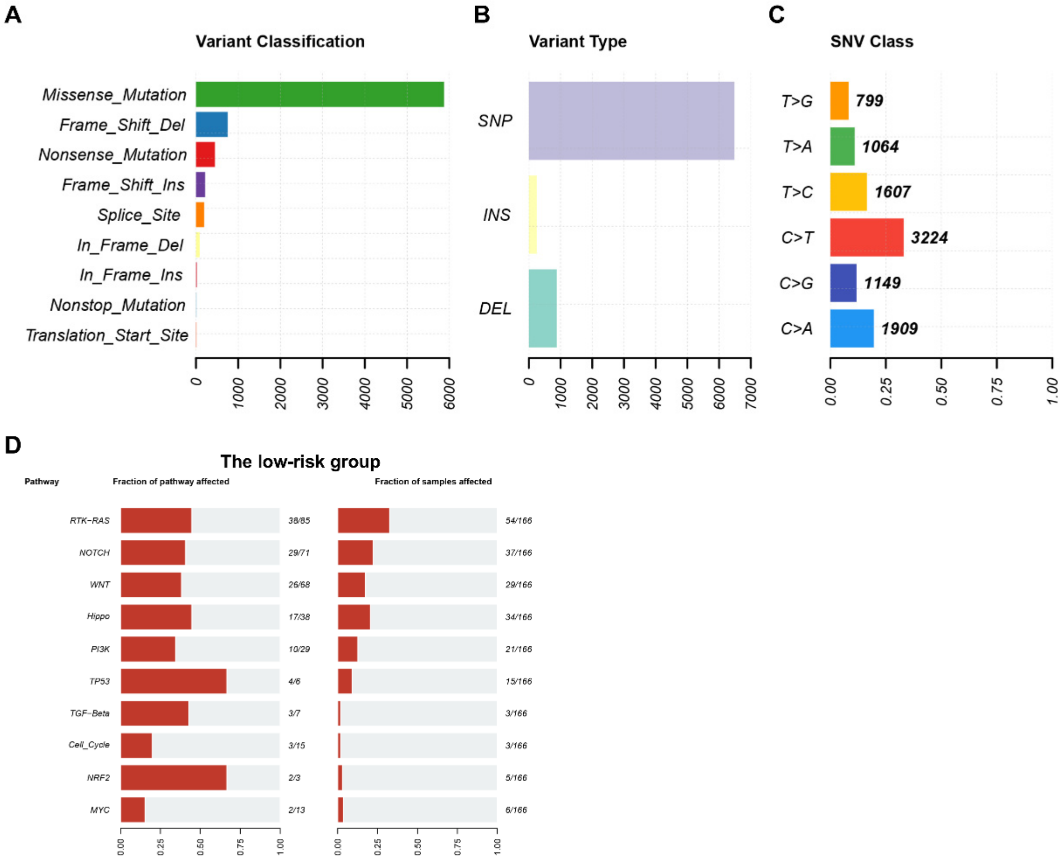

Supplemental Figure S5. CcRCC patients with high ENO2 expression have higher levels of tumor mutation burden (TMB)

(A-B) Gene mutation categories in the low-expression subgroup of ENO2. (C) Classification of SNVs in the low-expression subgroup of ENO2. (D) Top 10 signaling pathways enriched with mutated genes in the low-expression subgroup of ENO2.
